# Heart Failure in Acute Ischemic Stroke

**DOI:** 10.2174/157340310791658776

**Published:** 2010-08

**Authors:** Elisa Cuadrado-Godia, Angel Ois, Jaume Roquer

**Affiliations:** Neurology Department, Hospital Universitari del Mar. Program on Research on Inflammatory and Cardiovascular Disorders, IMIM, Barcelona, Spain

**Keywords:** Heart failure, stroke, outcome.

## Abstract

Heart failure (HF) is a complex clinical syndrome that can result from any structural or functional cardiac disorder that impairs the ability of the ventricle to fill with or eject blood. Due to the aging of the population it has become a growing public health problem in recent decades. Diagnosis of HF is clinical and there is no diagnostic test, although some basic complementary testing should be performed in all patients. Depending on the ejection fraction (EF), the syndrome is classified as HF with low EF or HF with normal EF (HFNEF). Although prognosis in HF is poor, HFNEF seems to be more benign. HF and ischemic stroke (IS) share vascular risk factors such as age, hypertension, diabetes mellitus, coronary artery disease and atrial fibrillation. Persons with HF have higher incidence of IS, varying from 1.7% to 10.4% per year across various cohort studies. The stroke rate increases with length of follow-up. Reduced EF, independent of severity, is associated with higher risk of stroke. Left ventricular mass and geometry are also related with stroke incidence, with concentric hypertrophy carrying the greatest risk.

In HF with low EF, the stroke mechanism may be embolism, cerebral hypoperfusion or both, whereas in HFNEF the mechanism is more typically associated with chronic endothelial damage of the small vessels. Stroke in patients with HF is more severe and is associated with a higher rate of recurrence, dependency, and short term and long term mortality. Cardiac morbidity and mortality is also high in these patients. Acute stroke treatment in HF includes all the current therapeutic options to more carefully control blood pressure. For secondary prevention, optimal control of all vascular risk factors is essential. Antithrombotic therapy is mandatory, although the choice of a platelet inhibitor or anticoagulant drug depends on the cardiac disease. Trials are ongoing to evaluate anticoagulant therapy for prevention of embolism in patients with low EF who are at sinus rhythm.

## INTRODUCTION

Heart failure (HF) is a major and growing public health problem in the United States, with approximately 5 million cases and over 550 000 patients diagnosed with HF for the first time each year [1]. HF is primarily a condition of the elderly, and thus the widely recognized “aging of the population” also contributes to its increasing incidence. Although patients with HF face a high risk of mortality and cardiovascular morbidity, survival has improved in recent years with better therapeutic strategies. Hospital discharges for HF rose from 877 000 in 1996 to 1 106 000 in 2006 [2] and the total estimated direct and indirect costs for HF have also increased, from $27.9 billion in 2005 to $39.2 billion in 2010 [3].

HF and ischemic stroke (IS) share similar risk factors and HF itself is a risk factor for IS. Moreover, IS in patients with HF is more severe and has a poor prognosis. The aim of this chapter is to review the main causes of HF, the risk of stroke in patients with HF, and the treatment and prognosis of HF in IS. 

## THE NATURAL HISTORY OF HEART FAILURE

Heart failure (HF) is a complex clinical syndrome that can result from any structural or functional cardiac disorder that impairs the ability of the ventricle to fill with or eject blood. HF is characterized by specific symptoms (dyspnea and fatigue) in the medical history and signs (edema, rales) during physical examination. 

The clinical syndrome of HF may result from disorders of the pericardium, myocardium, endocardium, or great vessels, but the majority of patients with HF have symptoms due to an impairment of left ventricle (LV) myocardial function. Coronary artery disease (CAD), hypertension, valvular disease and dilated cardiomyopathy are the main causes of HF in the western world [4]. Cardiomyopathies (CM) are a heterogeneous group of diseases of the myocardium associated with mechanical and/or electrical dysfunction; there is a variety of causes, frequently genetic. Inappropriate ventricular hypertrophy or dilatation is usually exhibited [5,6]. 

The classification system most commonly used to quantify the degree of functional limitation was developed by the New York Heart Association (NYHA). This system assigns patients to a functional class based on the degree of effort needed to elicit symptoms: Class I- symptoms of HF only at activity levels that would limit normal individuals; Class II- symptoms of HF with ordinary exertion; Class III- symptoms of HF with less than ordinary exertion; and Class IV- symptoms of HF at rest. 

However, there is a poor relationship between measures of cardiac performance and the symptoms produced by the disease. Patients with a very low EF may be asymptomatic, whereas patients with preserved LVEF may have severe disability. In fact, it is estimated that although 2.9% of the USA population has an EF≤30%, only half of these individuals have symptomatic HF [7]. 

HF is a progressive disorder. Its development has been characterized by 4 stages: Stage A- High risk for HF without structural heart disease or symptoms; Stage B- Heart disease with asymptomatic LV dysfunction; Stage C- Prior or current symptoms of HF; Stage D- Refractory end stage HF. In contrast to NYHA classification, this staging system emphasizes the progressive nature of HF and defines the appropriate therapeutic approach for each stage. 

The principal manifestation of the progression is a change in the geometry and structure of the LV, such that the chamber dilates and/or hypertrophies and becomes more spherical—a process referred to as cardiac remodeling. Cardiac remodeling generally precedes the development of symptoms (occasionally by months or even years), continues after the appearance of symptoms, and contributes substantially to worsening of symptoms despite treatment. The activation of endogenous neurohormonal systems plays an important role in cardiac remodeling and thereby in the progression of HF. 

Patients with HF have elevated circulating or tissue levels of norepinephrine, angiotensin II, aldosterone, endothelin, vasopressin, and cytokines, which can act (alone or in concert) to adversely affect the structure and function of the heart. These neurohormonal factors not only increase the hemodynamic stresses on the ventricle by causing sodium retention and peripheral vasoconstriction but may also exert direct toxic effects on cardiac cells.

Heart failure has been traditionally classified as “diastolic” (preserved EF) or “systolic” (reduced EF). However, this nomenclature has become the subject of controversy  [8] and some have suggested that the phenotypic expression of HF occurs on a continuum, with underlying myocardial dysfunction present in the early stages of the syndrome when diastolic abnormalities predominate  [9]. Since 2005 the American College of Cardiology-American Heart Association (ACC/AHA) guidelines for the diagnosis and management of heart failure have used the term “heart failure with preserved or normal ejection fraction” (HFNEF) rather than “diastolic heart failure”  [1]. Depending on the criteria used to delineate HF and the accepted cutoff for defining preserved LVEF, it is estimated that as many as 20% to 60% of patients with HF have a relatively normal LVEF and, in the absence of valvular disease, are believed to have reduced ventricular compliance as a major contributor to the clinical syndrome  [10,11]. In HFNEF, clinical presentation can be as dramatic as that in patients with low LVEF, for example in patients admitted with acute pulmonary edema  [12].

In general, a definitive diagnosis of HFNEF can be made when the rate of ventricular relaxation is slowed according to some diagnostic criteria  [13]. In practice, the diagnosis is generally based on the finding of typical symptoms and signs of HF in a patient who is shown to have a normal LVEF and no valvular abnormalities (aortic stenosis or mitral regurgitation, for example) on echocardiography. 

In the Acute Decompensated Heart Failure National Registry (ADHERE) database  [14], among 52 187 patients admitted to hospital for acute decompensated HF, patients with HFNEF (*n* = 26 322) were more likely to be older and female, and less likely to have CAD or a previous myocardial infarction. The HFNEF group was more likely to have comorbidities such as hypertension and diabetes than patients with HF and low EF. Atrial fibrillation (AF) and obesity have also been associated with HFNEF more frequently than HF with low EF  [10,15]. 

HF is the end stage of a cardiac disease and therefore prognosis is poor. The 1-year mortality rate for HF is high (20%) and 5-year survival is lower in men than in women: 59% of men and 45% of women will die within 5 years of HF diagnosis  [3].

The ADHERE registry  [14] also revealed a lower overall mortality in HFNEF patients compared with low EF patients, whereas symptom burden, duration of intensive care unit stay, overall length of hospital stay, and long-term mortality were similar between the two groups.

Most, but not all, series of patients with HFNEF have shown better survival than is seen in patients with HF and reduced LVEF  [10,11,16]. Moreover, secular trends have revealed slightly improved survival in patients with HF and low EF over the past 20 years, attributed to recent therapeutic advances in the treatment of systolic HF. Nevertheless, death rates from HFNEF remained unchanged.  [10,11,16] These studies highlight a change in HF epidemiology, with HFNEF prevalence increasing along with increasing age of the population, and the inadequacy of current therapeutic options for this disease.

## DIAGNOSIS

There are many ways to assess cardiac function. However, there is no diagnostic test for HF, since it is largely a clinical diagnosis based upon a physical examination and careful attention to the patient’s medical history. Recommendations for the evaluation of patients with HF are summarized in Table **I**. A complete history, including assessment of NYHA functional class, and physical examination are the first steps in evaluating the cause of HF and its severity. Direct inquiry may reveal prior or current evidence of MI, valvular disease or congenital heart disease, whereas examination of the heart may suggest the presence of cardiac enlargement, murmurs or a third heart sound. 

All patients with HF should have a 12-lead electrocardiogram (EKG), chest x-ray and complete laboratory analysis (blood count, urinalysis, serum electrolytes, glycohemoglobin, and blood lipids), as well as tests of renal, hepatic and thyroid function. Additional tests may be warranted to establish the etiology.

Measurement of natriuretic peptides (BNP, pro-BNP) in the emergency room helps to differentiate dyspnea due to HF from dyspnea due to other causes and reduces the length of hospitalization and the cost of treatment [17]. The single most useful diagnostic test in the evaluation of patients with HF is the 2-dimensional echocardiogram coupled with Doppler flow studies. Echocardiography has a high sensitivity and specificity for the diagnosis of myocardial dysfunction, and may also establish the etiology of HF  [18]. Other tests such as radionuclide ventriculography, magnetic resonance imaging or computed tomography might be useful in selected cases. 

Given that CAD is believed to be the underlying cause of HF with low EF in two-thirds of patients, all patients with unexplained HF should be evaluated for the presence of CAD. The noninvasive exercise test is a reasonable first step. In addition to detection of ischemic heart disease, exercise capacity can be used for risk stratification and determining prognosis as well as assessing the efficacy of therapy in patients over time. Coronary catheterization with angiography should be considered in patients with angina or a positive exercise test.

Angiography shows normal or near-normal coronary arteries in half of the patients with HF and low EF  [1]; most of these patients could have a myocardial disorder. MRI may be helpful in distinguishing IHD from CM and identifying the type. Endomyocardial biopsy and genetic evaluation should be reserved for patients with established indications.

## HF AND THE RISK OF ISCHEMIC STROKE

The risk of stroke in patients with HF has been related to the coexistence of vascular risk factors, the EF and left ventricle geometry. Although hypertension is the strongest risk factor for stroke, epidemiological studies have found increased risk of stroke associated with other risk factors, including cardiovascular abnormalities such as CAD, HF and atrial fibrillation (AF)  [19]. In the Framingham study, the age-adjusted 2-year incidence of stroke was more than double in the presence of CAD, more than triple in the presence of hypertension, more than quadruple in the presence of HF and nearly quintupled when AF was present  [19]. 

AF and HF often coexist in the same patient. AF is present in 10–50% of patients with HF, with the highest incidence in those with NYHA functional class IV; HF itself is also an independent predictor of AF  [20]. In a meta-analysis of independent risk factors for stroke in patients with AF, the associated variables were prior stroke/TIA (RR: 2.5), increasing age (RR: 1.5 per decade), history of hypertension (RR: 2.0) and diabetes mellitus (RR: 1.7). Clinical HF was not consistently an independent predictor in AF patients; some studies found an association in patients with low EF that has not been confirmed in other studies  [21].

Vascular risk factors (VRF) that increase the risk of stroke in AF (prior stroke, age, hypertension and diabetes) appear to also increase the stroke risk in HF, but the published findings are inconsistent. Age, prior stroke and diabetes were found to be risk factors in a community based study that compared stroke risk in 630 HF patients with the general population  [22]. However, the Studies of Left Ventricular Dysfunction (SOLVD), which included 6738 patients with symptomatic or asymptomatic HF, found that age, hypertension and prior stroke were risk factors only in men. In women, diabetes and smoking were the only VRF.  [23] Other retrospective studies have found only age  [24] or hypertension  [25] to be significant VRF of stroke in patients with HF. 

Persons with HF have higher incidence of strokes; nevertheless, there is marked variability in the stroke rates reported in different studies. As would be expected, the stroke rate in unselected cohorts of persons with HF is slightly higher than in clinical trials. Indeed, stroke rate varies from 1.7% to 10.4% in cohort studies  [26-28] compared with 0.6% to 4% in HF clinical trials  [23,24,29-31]. The heterogeneity in study design and follow-up periods makes it difficult to generalize these findings to clinical practice. A recent meta-analysis of 26 population studies of chronic HF with IS during follow-up, irrespective of EF or heart rhythm, found a stroke rate (95%CI) of 18.4 [16.9-19.9) per 1000 cases during the first year of HF. This rate tended to increase with duration of follow-up, to a maximum of 47.4 [45.6-49.2) strokes per 1000 cases at 5 years. In this meta-analysis, studies with a higher proportion of men, those conducted in 1990 or earlier, and cohort studies reported higher stroke rates than studies with more women, those conducted after 1990 and clinical trials  [32].

In community studies, the prevalence of stroke/TIA is higher in individuals with HF than in the general population. In the Framingham study, the adjusted risk of stroke associated with HF was 4.3 at 2 years follow-up  [19]. In a study in Olmsted County, the stroke risk among those with HF was 2.9 times the control population risk over 5 years  [22]. In the Reasons for Geographic And Racial Differences in Stroke (REGARDS), a U.S. population study that has included more than 30 000 participants, the prevalence of self-reported history of stroke or TIA was analyzed for HF, defined by the use of digoxin. The adjusted OR for the association between HF and stroke/TIA was 3.0 (95% CI: 2.2-4.0) [33].

Reduced EF (symptomatic or asymptomatic) has been found to be a risk factor for stroke, most often in prospective studies of MI survivors. In general, the stroke rate is relatively low, ranging from 1.3% to 3.5% per year. However, stroke was not the primary endpoint and these studies might have underestimated the occurrence [34] Survival and Ventricular Enlargement (SAVE) was a prospective trial with 5 years follow-up that assessed the relation between LVEF and the incidence of stroke in 2231 patients with LV dysfunction after acute MI but without symptomatic HF  [24]. LVEF was found to be an independent risk factor. For every 5% decrease in EF there was an 18% increase in the risk of stroke. In addition, patients with EF lower than 28% had a relative risk of stroke of 1.86, compared with patients with higher LVEF. Moreover, retrospective analysis of SOLVD data found a 58% increase in risk of thromboembolic events for every 10% decrease in EF among women. There was no significant increase in stroke risk among men in that trial  [23]. Low EF was a risk factor for stroke in the multiethnic North Manhattan (NOMASS) population cohort, independently of age, sex and ethnicity; however, risk of stroke was not related to severity of EF reduction [35]. 

The TOAST (Trial of Org 10172 in Acute Stroke Treatment) classification [36], the most widely accepted etiological classification of ischemic strokes, was actualized in 2005 by the Stop Stroke Study TOAST (SSS-TOAST) system [37]. Due to new epidemiological evidence, symptomatic HF with low EF and chronic myocardial infarction (MI) with EF less than 28% were included as primary high risk sources of embolic stroke. Recent MI (< 4 weeks) and dilated CM were in the high risk category in the original classification and were maintained in the new system. All sources of cardiac embolism considered in this classification are listed in Table **II**. High risk cardiac sources have greater than 2% annual primary risk for stroke. The low-risk group includes cardiac sources with less than 2% primary risk for stroke, yet some cardiac abnormalities associated with increased risk for recurrent stroke but undetermined risk for first-ever stroke were also included in this category.

In patients with non-ischemic dilated CM, the rate of stroke appears similar to that in patients with CM due to CAD. Again there is high variability across studies and most of the studies included small series of patients  [38,39]. 

Apart from systolic function or EF, LV mass and geometry are also related with the emergence of strokes. Symptomatic or asymptomatic increased LV mass, known as LV hypertrophy (LVH), have proved to be a risk factor for cardiovascular morbidity and mortality, including ischemic stroke  [40-43]. Increased stroke with LVH risk has been shown in all race-ethnicities  [35,40,43-45] and the risk is independent of other cardiovascular risk factors, including arterial hypertension  [46]. This association between LVH and IS has been identified across studies using ECG [41,42], echocardiography  [47,48] and MRI  [49]. Some studies have demonstrated that patients who fail to reduce LVH are much more likely to suffer cardiovascular events, including stroke, than those in whom the LV mass is reduced by antihypertensive treatment  [50-54] or physical activity [55].

Moreover, abnormal LV geometry beyond the simple LV mass increase has also been associated with an increased risk of cardiovascular morbidity. LV geometry may be classified into the following four mutually exclusive groups on the basis of LV mass and relative wall thickness (RWT): 1) concentric hypertrophy (increased mass and increased RWT), 2) eccentric hypertrophy (increased mass and normal RWT), 3) concentric remodeling (normal mass and increased RWT) and 4) normal geometry (normal mass and normal RWT). Concentric hypertrophy carries the highest risk, followed by eccentric hypertrophy  [56,57]. The role of concentric remodeling is more controversial, having conferred cardiovascular risk beyond LV mass in some studies but not in others  [58-61]. 

Several studies have assessed the specific risks of LVH and LV geometry in the development of IS. In a case-control study from the multiethnic NOMASS cohort, LVH was associated with a 2.5-fold increase in stroke risk after adjustment for other stroke risk factors across all the racial and ethnic subgroups. Concentric hypertrophy carried the greatest stroke risk, followed by eccentric hypertrophy and concentric remodeling  [48]. In the Multi-Ethnic Study of Atherosclerosis (MESA)  [49], the relationship of LV mass and geometry, measured with cardiac MRI, to incident cardiovascular events was analyzed in 5,098 participants. Increased LV mass-to-volume ratio was independently associated with stroke (HR: 4.2 per g/ml, p = 0.005). In contrast, LV mass showed the strongest association with incident HF events (HR: 1.4 per 10% increment, p < 0.0001). The MESA findings point out the importance of LV geometry for the emergence of IS compared to other cardiovascular diseases.****

## MECHANISMS OF ISCHEMIC STROKE IN HF

Under the current TOAST criteria for stroke subtype,  [37] the mechanism of stroke in patients with HF and low EF is classified as cardiogenic embolism if other etiologies are ruled out. However, the stroke mechanism in HF may be embolism or cerebral hypoperfusion. LV dysfunction causes an increased LV end diastolic volume that promotes blood stasis in both the LV and left atrium, increasing the chance of thrombus formation and the risk of embolic stroke. About 12% of patients with CM have LV thrombus formation and EF is the factor most associated with ventricular thrombus formation  [62]. A variety of factors associated with HF predispose to thrombosis. These include vascular pathology, increased coagulability and impaired flow. Several studies have shown that patients with HF have increased plasma concentrations of fibrinopeptide A, D-dimer, von Willebrand factor, fibrinolytic products, beta-thromboglobulin and endothelial procoagulants  [63]. These hemostatic abnormalities that predispose to thromboembolic events have been associated with the neuroendocrine activation  [64]. 

However, stroke in HF might be also due to cerebral hypoperfusion. Patients with low EF have an elevated LV filling pressure and a reduced stroke volume, and this causes a reduction of systemic blood flow. In patients with adequate cerebrovascular reactivity, lowering cerebrovascular resistance through dilatation of the brain arterioles could compensate for the reduced cardiac output. Autoregulation maintains cerebral blood flow through a wide range of systemic blood pressure. Therefore, a reduction in EF should not affect cerebral blood flow in a patient with intact autoregulation. However, patients with HF may easily decompensate hemodynamically and may become hypotensive secondary to cardiac ischemia, arrhythmia or over-medication with hypotensive drugs. This would limit the potential for further dilatation, resulting in the altered cerebrovascular reserve capacity observed in patients with HF. Increasing severity of HF, indicated by NYHA grade and decreasing EF, are correlated with decreased cerebrovascular reactivity  [65] and decreased global cerebral blood flow  [66]. 

In a case/control study, HF was associated with prior stroke/TIA. The risk was higher in patients in the lowest tertile of systolic blood pressure (SBP)  [33]. Vulnerability of the brain to hypoperfusion in HF is also supported by the increased risk of cognitive impairment in HF patients and low SBP  [67]. These findings have raised the question whether SBP must be reduced to the lowest level tolerated, as has been recommended in patients with HF  [33]. 

Embolic and hypoperfusion related strokes differ in their localization. Embolic strokes more frequently affect carotid than vertebral circulation, due to the different pathways of blood supply. In terms of carotid circulation, MCA is more affected than ACA. Embolic infarcts are typically medium or large size and located in the cortical areas of the brain, although subcortical strokes might occur in the case of a small embolus that occludes a small perforant artery  [68]. Fig. (**1**) is an example of embolic stroke.

**Fig. (2):**
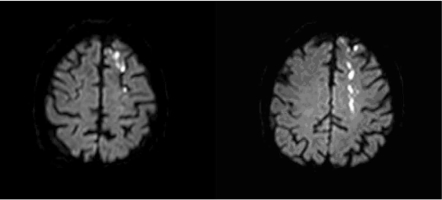
MRI-DWI showing a watershed infarct between left MCA-ACA territories.

In hypoperfusion related ischemia, three types have been described as the most frequent  [69]:

1. Cortical watershed infarcts (Fig. **2**), located at the junction of the usual territories of major cerebral arteries in free anastomosis (anterior, middle, and posterior cerebral);
2. Distal field infarcts, confined to a location at the most distal field of supply of an artery that has few or no collaterals, such as the upper lateral margin of the lateral ventricle (distal field of the lenticulostriate arteries); and
3. Internal border-zone infarcts, deep infarcts that lie between the territories of 2 arteries that do not freely anastomose, such as white matter infarcts lying between territories supplied by long penetrators of the anterior, middle, or posterior cerebral artery.

Nevertheless, hypoperfusion and embolism can coexist and reduced perfusion may exacerbate ischemia resulting from embolism  [70]. In a case-control study, MRI infarct volume was measured in a cohort of patients with EF less than or equal to 35% with or without concomitant carotid stenosis less than 70% and compared with controls with normal EF. Patients with reduced EF tended to have larger cortical infarcts than patients with normal EF. The mean volume of infarcts in patients with high-grade carotid stenosis in addition to low EF was greater than in patients with low EF, suggesting a hemodynamic interaction between arterial stenosis and reduced cardiac output  [69]. 

Nevertheless, the mechanism of stroke in patients with HFNEF is not clear. LV hypertrophy might be a marker of sub clinical disease that predisposes to other conditions directly involved in stroke etiology. Hypertension is well recognized as the most frequent antecedent of left ventricular hypertrophy in the general population  [71] and is the leading risk factor for cerebrovascular disease  [72]. Moreover, LV mass correlates with carotid wall thickness and carotid plaques  [53,73], which are other important risk factors for cerebrovascular disease  [74,75]. Among patients with systemic hypertension, arterial structure and function are most abnormal when concentric LV hypertrophy is present and may contribute to the greater risk for ischemic strokes associated with this geometric pattern [49].

Concentric hypertrophy and concentric remodeling have been more associated with symptomatic and asymptomatic lacunar strokes.  [48, 76], while eccentric hypertrophy is more associated with an excess of cardioembolic strokes  [48]. Lacunar infarcts or small subcortical infarcts result from occlusion of a single penetrating artery, usually in patients with previous history of hypertension or diabetes. Lacunar infarcts show a paradoxical clinical course with a favorable prognosis in the short term, with low mortality and low disability at hospital discharge but with an increased risk of death, stroke recurrence and dementia in the mid and long term [77]. Progression of the small vessel disease is believed to be caused by arteriolar endothelial dysfunction facilitated by hypertension and diabetes, which would increase permeability of the brain blood barrier with extravasation of toxic substances (mainly proteases), producing neuronal and glial damage [78]. Therefore, endothelial dysfunction may be the link between vessel thickening and heart remodeling. 

## HF AND OUTCOME IN ISCHEMIC STROKE

The long-term prognosis for survival free of recurrent stroke is important to patients and their caregivers and clinicians. Cardioembolic strokes have been associated with the most severe acute neurological deficit and the highest mortality and worst functional outcome at 90 days [79, 80]. In fact, in the EUROSTROKE study  [41], LVH in the ECG was associated with an increased risk of fatal stroke, whereas risk for non-fatal stroke was non-significant. In addition, patients with low EF had more severe strokes as measured by National institute of Health Stroke Scale (NIHSS>6) than patients with normal EF in the NOMASS study [35]. 

Several community-based studies of stroke and TIA prognosis have analyzed the short- and long-term predictors of death and disability. Short-term mortality (30-90 days) has been related to age, stroke severity, congestive HF, and AF  [81-84]. For mortality at 1 and 5 years, age, stroke severity and congestive HF continue to predict mortality as well as onset of ischemic heart disease  [82,85-87]. 

In a community-based study that prospectively included 377 patients with first-ever IS during 12 months HF, stroke severity (NIHSS≥6) and mortality at 28 days were associated with AF and previous dementia  [88]. The same cohort of patients was followed up within 12 months in a later study that analyzed the predictors of survival, with dependency defined as a modified Rankin Scale (mRS) [89] greater than 2 and stroke recurrence. Dementia, age, stroke severity and AF were associated with death within 1 year. HF was independently associated with 1-year dependency (adjusted OR: 3.0) but not with survival or stroke recurrence  [90]. These findings suggested that patients with HF who have an incident stroke have a high risk of early mortality or mid-term dependency due to the index stroke severity. Nevertheless, HF seems to be also a strong predictor of long-term mortality. In a recent study analyzing the predictors of 5-year mortality in a large cohort of young patients (age 15 to 49) with first-ever IS, HF defined as EF<55% was found to be second only to active malignancies at the time of the stroke in its association with mortality (adjusted OR: 5.25)  [91].

In most of the previous studies HF has been defined as a clinical syndrome without echographic information. Our group analyzed the effect of both forms of HF (with low EF and HFNEF) on the 90-day outcome in 540 consecutive patients with IS from the BASICMAR cohort  [81] studied with echocardiogram  [92]. We found an independent association between both forms of HF and poor outcome, defined as mRS 3-6 at 90 days, a composite outcome that includes dependency and mortality. The adjusted OR was higher for HF with low EF (adjusted OR: 3.01, p=0.008) than HFNEF (adjusted OR: 2.53, p < 0.001). Initial stroke severity was also independently associated with poor outcome, whereas thrombolytic treatment and statin pretreatment were protective factors. HFNEF patients were older, more frequently women, and less likely to be current smokers than patients with low EF. However, there were no differences in initial NIHSS neurological severity between HFNEF and HF with low EF  [93].

The poor prognosis in cardioembolic stroke is believed to be caused by the index stroke because early stroke recurrence seems to be low in these patients. Nevertheless, in a study of the BASICMAR cohort we analyzed the potential risk factors for 7- and 90-day recurrence in patients with minor stroke and TIA. We found that HF was independently associated with stroke recurrences at 7 days (adjusted OR: 2.66) and at 90 days (2.41)  [94]. In the NOMASS cohort, HF was not independently associated with stroke recurrence at 30 days, 1 year or 5 years. However, the recurrent stroke rate was notably higher in patients with HF (mean 44%) compared with patients without HF (25%)  [95].

The explanation for a worse prognosis, independent of EF, in stroke patients with history of HF may be related to the main underlying causes such as CAD, hypertension, and valvular disease. These patients have a higher atherosclerotic burden and endothelial dysfunction, factors proven to be associated with higher mortality [96-98]. Moreover, older age of patients with prior HF may be a contributory factor, especially in HFNEF [90,92].

Most deaths after IS can be directly attributed to the initial neurological injury. However, approximately 2-6% of all stroke patients die from cardiac causes in the 3 months after IS  [99]. After the acute period, cardiac risk declines but remains higher than for age-matched controls. The estimated annual rate of MI and non-stroke vascular death in stroke patients is 2.2% and 2.1%, respectively. The accumulation of risk is linear, suggesting that at 10 years after an IS, the risk would be 20%  [100]. In a large study conducted to analyze the prognostic determinants for cardiac morbidity and mortality within 3 months after IS, the most predictive factor was history of HF (OR:3.33), followed by diabetes (OR: 2.11), baseline creatinine (OR:1.77), severe stroke (OR:1.98) and long QTc or ventricular extrasystoles on ECG (OR: 1.93) [101]. As a cause of death in the acute IS population, cardiac mortality was second only to neurologic death directly resulting from the incident stroke.

It is not infrequent that patients with acute strokes show an increase in the concentration of cardiac troponin, BNP and NT-proBNP  [102,103]. Moreover, patients with higher concentration of these biomarkers have a worse prognosis  [104]. Although there are a number of possible causes of myocardial damage and raised troponin after a stroke, the mechanism is likely to be the activation of a rennin angiotensin aldosterone system (RAAS), which leads to vasoconcentration and fluid retention, followed by myocardial stress. The myofibrillar necrosis occurs in the vicinity of the cardiac nerves, and not in the macrovascular distribution seen in patients with CAD  [105]. Neurogenic damage is also responsible for cardiac stunning, defined as a transient decrease in cardiac function associated with ST and Q wave abnormalities and segmental hypokinesis that resolves a few days after the onset of the symptoms. It is believed to reflect transient coronary vasospasm secondary to increased sympathetic tone [106]. Patients with HF and chronic neurohormonal activation might have poor adaptability following a stroke, with peripheral hypoperfusion and more myocardial damage, leading to higher mortality. 

## TREATMENT CONSIDERATIONS IN HF PATIENTS WITH IS

Patients with HF that experienced an IS should be treated according to the current guidelines  [107]. Treatment of stroke has different phases, including primary prevention, acute phase, rehabilitation, and secondary prevention. As previously discussed, patients with HF have a high risk of IS. Therefore, careful VRF control and optimization of cardiac output with maintenance of normal blood pressure and a normal heart rate is essential in these patients. 

Acute phase treatment includes revascularization (pharmacologic or mechanical thrombolysis, angioplasty) and general management. HF patients would benefit from acute treatment in a stroke unit, which has proven to lower the risk of early neurological deterioration, improve the outcome and decrease the length of stay for stroke patients [108]. 

Blood pressure treatment during the acute phase is a controversial area in stroke management and different clinical trials are ongoing  [109]. Patients with the highest and lowest levels of blood pressure in the first 24 hours after stroke seem to be more likely to have early neurological decline and poorer outcomes; in general, mild hypertension is preferred during the first 24h. In patients with HF, blood pressure can usually be raised by adequate rehydration with saline solutions; patients with low cardiac output may occasionally need inotropic support. Rhythm control is also essential to optimize cardiac function.

Secondary prevention includes strict management of all cardiovascular risk factors. In the AHA/ASA guidelines  [110] for secondary prevention in stroke patients, antihypertensive treatment is recommended for prevention of recurrent stroke and other vascular events in persons who have had an ischemic stroke or TIA and are beyond the hyperacute period (Class I, Level of Evidence A). Normal BP levels have been defined as <120/80 mm Hg that coincide with the ACCF/AHA recommendations for HF patients.

This is particularly appropriate in patients with HFNEF, whose symptoms may respond particularly well to treatments that lower blood pressure  [111].

The optimal drug regimen for BP management remains uncertain and the choice of specific drugs must be individualized by specific patient characteristics. In fact, a recent meta-analysis of randomized trials that tested blood pressure lowering agents on stroke recurrence in patients with stroke/TIA found that all agents that managed to lower the blood pressure also reduced recurrent stroke and cardiovascular events  [112].

Therefore, drugs that can both control BP and treat HF should be preferred. This includes the use of diuretics, ACEIs, and beta blockers. However, some antihypertensive agents should be avoided in patients with HF because of their ability to depress cardiac function, such as most calcium channel blockers, or lead to salt and water retention, such as minoxidil. 

Increased sympathetic activity has been found after acute stroke and is associated with poor neurological prognosis. Beta-blocker use has been associated with less severe strokes on presentation and may be cerebroprotective due to a sympatholytic effect and inhibition of thrombin generation, modulation of blood glucose and reduction of acute inflammation  [113]. Therefore, use of one of the beta-blockers proven to reduce mortality (bisoprolol, carvedilol and sustained release metoprolol succinate) is recommended for IS patients with stable HF  [1].

HF has been associated with resistance to the actions of insulin  [114] and the resulting hyperinsulinemia may promote both cardiac and vascular hypertrophy, hastening the progression of HF  [115]. Moreover, diabetes mellitus is a risk factor for stroke recurrence  [116] and strict diabetes control is mandatory in these patients.

The drugs routinely used in the management of HF in nondiabetic patients are also administered to those with diabetes mellitus. ACEI and beta-blockers prevent the progression of HF in diabetic and non diabetic patients. However, thiazolidinediones have been associated with increased peripheral edema and symptomatic HF and these oral antidiabetic agents should be used with caution in diabetic patients. 

AF might worsen HF symptoms and increase the risk of thromboembolic events by several mechanisms, including compromising cardiac output, decreasing coronary perfusion and aggravating cardiac contraction and relaxation. Control of ventricular rate and prevention of thromboembolic events are essential elements of treatment in patients with HF and AF. However, rhythm control has not been found to be more effective in preventing cardiovascular deaths or embolic events including strokes compared with rate control strategy in patients with HF and AF  [20]. The rate-control therapy reduces rates of hospitalization and need for repeated cardioversion and is recommended in these patients. 

### Antithrombotic Therapy

In general, patients with cardiac disease and cerebral infarction face a high risk of recurrent stroke. Because it is often difficult to determine the precise mechanism, the choice of a platelet inhibitor or anticoagulant drug may be difficult. Moreover, trials studying the stroke risk or response to anticoagulation have included patients with CM, low EF and HF interchangeably  [117].

In the AHA/ASA Guidelines  [110] for secondary prevention of stroke, recommendations for medical treatment in patients with cardiogenic embolism include the following: 

For patients with ischemic stroke or TIA who have dilated CM, either warfarin (INR 2.0-3.0) or antiplatelet therapy may be considered for prevention of recurrent events (Class IIb, Level of Evidence C). Potential antiplatelet therapies used to prevent recurrent stroke include aspirin (50-325 mg/d), the combination of aspirin (25 mg twice daily) and extended release dipyridamole (200 mg twice daily), and clopidogrel (75 mg daily). However, IS in patients with HF due to AF, acute MI with LV thrombus, rheumatic mitral valve disease or prosthetic heart valves should be treated with oral anticoagulants. 

Nevertheless, there is no clear indication of anticoagulation in patients with HF, without valvular disease, that are in sinus rhythm. The current guidelines from the AHA/ACC  [118] do not support the routine use of warfarin in patients with dilated CM. In the European Stroke Initiative recommendations for stroke management  [107], there is no specific recommendation for primary or secondary stroke prevention in patients with cardiomyopathy who are at sinus rhythm. There are also no data on whether anticoagulation is beneficial in patients with HFNEF. 

In the SAVE study, both warfarin and aspirin (given separately) were associated with a lower risk for stroke than no antithrombotic therapy  [24]. Other large studies of MI patients have compared the effect of warfarin and aspirin in the prevention of new vascular events. Results from these trials show that warfarin, at moderate or high dose (INR 2.8 to 4.8), in combination with aspirin or given alone, is superior to aspirin alone in reducing the incidence of composite events after an acute MI but is associated with a higher risk of bleeding. However, warfarin at low dose (INR <2) plus aspirin is not superior to aspirin (80mg) alone  [119].

Warfarin appears to exert a similar effect on the reduction of stroke both in patients with nonischemic CM and in those with ischemic heart disease  [38]. Therefore, warfarin is prescribed to prevent cardioembolic events in patients with CM, although no randomized clinical studies have demonstrated the efficacy of anticoagulation. Several trials have been initiated to address this issue.

The Warfarin and Antiplatelet Therapy in Chronic Heart Failure Trial (WATCH)  [120] was designed to evaluate the efficacy of antithrombotic strategies among symptomatic HF patients in sinus rhythm with EF ≤ 35%. Patients were randomized to open-label warfarin (target INR 2.5 -3.0) or double-blind antiplatelet therapy with aspirin (162 mg) or clopidogrel (75 mg). The trial was terminated early for poor recruitment after 1587 patients of the 4500 planned were enrolled, with a resulting reduction of its power to achieve its original objective. The primary outcome was the time to first occurrence of death, nonfatal myocardial infarction, or nonfatal stroke. For the primary composite endpoint, the hazard ratios were as follows: for warfarin vs. aspirin, 0.98 ,p=0.77; for clopidogrel vs. aspirin, 1.08, p=0.57; and for warfarin vs. clopidogrel, 0.89, p=0.39. However, warfarin was associated with fewer nonfatal strokes than aspirin or clopidogrel. 

The ongoing Warfarin Versus Aspirin for Reduced Cardiac Ejection Fraction (WARCEF) study  [121] is comparing primary endpoint (time to first occurrence of IS, intracerebral hemorrhage or death) between warfarin (INR 2.5-3) and aspirin (325 mg) in patients in all NYHA classes (I-IV) with low EF (≤ 35%) who are at sinus rhythm. The target enrollment is 3201 patients. This trial is not statistically powerful enough to determine whether warfarin has an effect on ischemic stroke risk reduction; however, by pooling results with those of other trials, we may be able to draw some conclusions about this issue. 

## CONCLUSIONS

Heart failure is a growing health problem due in part to population aging. Heart failure and stroke share similar risk factors and heart failure is associated with higher risk of IS, especially in patients with low EF. The stroke mechanisms include embolism and hypoperfusion. Stroke in HF is associated with more severity, recurrence risk and early- and long-term mortality regardless of EF. Current studies on new acute phase treatments and secondary prevention have the potential to improve the prognosis.

## Figures and Tables

**Fig. (1):**
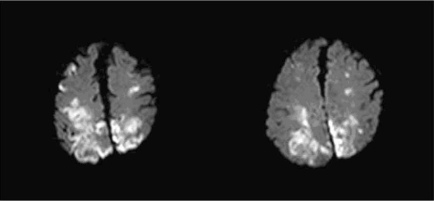
MRI Diffusion-weighted image (DWI) showing bilateral infarcts in MCA and ACA territories suggestive of embolic origin.

**Table I:** Recommendations for the Evaluation of Patients with HF Included in the 2005 ACC/AHA Guidelines with 2009 Focused Update (1)

**Class I - There is Evidence and/or General Agreement that the Initial Evaluation of Patients Presenting with HF Should Include the Following:**
A complete history and physical examination to identify cardiac and non cardiac disorders or behaviors that might cause or accelerate the development or progression of HF.
A careful history of current and past use of alcohol, illicit drugs, standard or "alternative" therapies, and chemotherapy drugs.
An assessment of the ability to perform routine and desired activities of daily living.
An assessment of the volume status, orthostatic blood pressure changes, height and weight, and calculation of body mass index.
Laboratory studies including complete blood count, urinalysis, serum electrolytes (including calcium and magnesium), blood urea nitrogen, serum creatinine, fasting blood glucose (glycohemoglobin), lipid profile, liver function tests, and serum thyroid-stimulating hormone.
A 12-lead electrocardiogram and chest radiograph (posteroanterior and lateral).
Two-dimensional echocardiography with Doppler to assess left ventricular ejection fraction (LVEF), left ventricular size, wall thickness, and valve function. Radionuclide ventriculography can be performed to assess LVEF and volumes.
Coronary arteriography if there is a history of angina or significant ischemia unless the patient is not eligible for revascularization of any kind.
**Class IIa - The Weight of Evidence or Opinion is in Favor of Benefit From Performing the Following Studies as Part of the Initial Evaluation of Patients Presenting with HF:**
Coronary arteriography in patients who have chest pain that may or may not be of cardiac origin who have not had a prior evaluation of their coronary anatomy and are eligible for coronary revascularization.
Coronary arteriography in patients with known or suspected coronary artery disease who do not have angina and are eligible for revascularization.
Noninvasive imaging to detect myocardial ischemia and viability in patients with known or suspected coronary artery who do not have angina and are eligible for revascularization.
When the contribution of HF to exercise limitation is uncertain, maximal exercise testing with or without measurement of respiratory gas exchange and/or blood oxygen saturation.
To identify candidates for cardiac transplantation or other advanced treatments, maximal exercise testing with measurement of respiratory gas exchange.
In selected patients, screening for hemochromatosis, sleep disturbed breathing, or human immunodeficiency virus (HIV) infection.
When suspected clinically, diagnostic tests for rheumatologic disease, amyloidosis, or pheochromocytoma.
Endomyocardial biopsy when a specific diagnosis is suspected that would influence therapy.
Measurement of serum B-type natriuretic peptide (BNP) in the urgent care setting if the clinical diagnosis of HF is uncertain. Measurement of natriuretic peptides (BNP and NT-proBNP) can be useful in risk stratification.
**Class IIb - The Weight of Evidence or Opinion is Less Well Established for the Following Testing in the Initial Evaluation of Patients with HF**
Noninvasive imaging to define the likelihood of coronary artery disease in patients with left ventricular dysfunction.
Holter monitoring in patients who have a history of myocardial infarction and are being considered for electrophysiologic study to document the inducibility of ventricular tachycardia.
**Class III - There is Evidence and/or General Agreement that the Following Tests are not Useful or may be Harmful in the Initial Evaluation of Patients with HF**
Routine endomyocardial biopsy in the absence of suspicion of a specific diagnosis that would influence therapy suspected.
Routine signal-averaged electrocardiography.
Routine measurement of serum neurohormones other than BNP (eg, norepinephrine or endothelin).

**Table II:** Cardioaortic Sources of Cerebral Embolism According to the SSS TOAST Classification [37]

**Sources with High Primary Risk for Ischemic Stroke**

*Sources of embolism of thrombotic origin*
-Left atrial thrombus
-Left ventricular thrombus
-Atrial fibrillation
-Paroxysmal atrial fibrillation
-Sick sinus syndrome
-Sustained atrial flutter
-Recent myocardial infarction (within 1 month)
-Rheumatoid mitral or aortic valve disease
-Bioprosthetic and mechanical heart valves
-Chronic myocardial infarction together with low ejection fraction less than 28%
-Symptomatic congestive heart failure with ejection fraction less than 30%
-Dilated cardiomyopathy
-Nonbacterial thrombotic endocarditis
*Sources with embolism not predominantly of thrombotic origin*
-Infective endocarditis
-Papillary fibroelastoma
-Left atrial myxoma

**Sources with Low or Uncertain Primary Risk for Ischemic Stroke**

*Cardiac sources of embolism*
-Mitral annular calcification
-Patent foramen ovale
-Atrial septal aneurysm
-Atrial septal aneurysm and patent foramen ovale
-Left ventricular aneurysm without thrombus
-Isolated left atrial smoke (no mitral stenosis or atrial fibrillation)
*Aortic sources of embolism*
-Complex atheroma in the ascending aorta or proximal arch
